# Mitochondria and peroxisomes: partners in autophagy

**DOI:** 10.1080/15548627.2022.2155368

**Published:** 2022-12-26

**Authors:** Léa P. Wilhelm, Ian G. Ganley

**Affiliations:** MRC Protein Phosphorylation and Ubiquitylation Unit, University of Dundee, Dundee DD1 5EH, UK

**Keywords:** Autophagy, mitophagy, pexophagy, BNIP3L, NIX

## Abstract

Mitochondria, often called “the powerhouse” of the cell due to their role as the main energy supplier, regulate numerous complex processes including intracellular calcium homeostasis, reactive oxygen species (ROS) production, regulation of immune responses, and apoptosis. So, mitochondria are a fundamental metabolic hub that also control cell survival and cell death. However, they are not unique in all these functions. Indeed, peroxisomes are small cytoplasmic organelles that also ensure metabolic functions such as fatty acid oxidation and ROS production. This common relationship also extends beyond function as peroxisomes themselves can form from mitochondrial-derived precursors. Given this interconnection between mitochondria and peroxisomes involving biogenesis and function, in our recent work we determined if their turnover was also linked.

Macroautophagy is a major route for the specific turnover of organelles, including mitochondria and peroxisomes (termed mitophagy and pexophagy, respectively). Much is known about the mechanisms of mitophagy; however less is known, at least in mammalian systems, on the control of pexophagy. Two types of signals that prime mitochondria for mitophagy have now been described. First, the so-called “eat-me” priming signal can consist of ubiquitination of outer mitochondrial membrane (OMM) proteins, present at the mitochondrial surface. This signal is recognized by the sequestosome-like receptors (SLRs), including SQSTM1/p62, NBR1, CALCOCO2/NDP52, TAX1BP1, and OPTN. These all contain ubiquitin-binding domains as well as LC3-interacting regions (LIRs) or RB1CC1/FIP200 binding motifs that directly interact with autophagy machinery. Thus, the SLRs act as bridges to link ubiquitinated mitochondria to the autophagic machinery and the forming autophagosome. The second type of mitophagy signal is SLR-independent and bypasses the need for a ubiquitination priming signal. Here, specific OMM proteins themselves act as receptors, which are capable of interacting directly with the autophagy machinery. Multiple OMM proteins have been identified as mitophagic receptors, including BNIP3, BNIP3L and FUNDC1, all of which contain a LIR at their N-terminal region that interacts with Atg8-family proteins on the forming autophagosome. As with mitochondria, peroxisomes can also be turned over via a ubiquitin- and SLR-dependent mechanism. However, much less is known about whether SLR-independent pexophagy exists for mammalian peroxisomes.

Given the above, in our recent work [1], we started to explore the relationship between mitophagy and pexophagy in more detail. We set out to address several questions: Are peroxisomes turned over under the same conditions as mitochondria? Can mitophagy and pexophagy happen at the same time? Does inhibition of one increase the other? Are they regulated by the same signaling pathway?

First, we found that multiple selective autophagy pathways can be activated under the same conditions. By using biochemistry as well as fluorescent mitophagy and pexophagy reporters (*mito-*QC and *pexo*-QC, respectively), we established that mitochondria and peroxisomes are lysosomally turned over following iron chelation by deferiprone (DFP) or hypoxia. Turnover of both requires the HIF1A transcription factor, which itself is stabilized under these treatments. Second, our results show that BNIP3L/NIX, previously described as an SLR-independent mitophagy receptor that is induced under hypoxic conditions, is also required for pexophagy. In BNIP3L-depleted cells, not only is DFP-induced mitophagy impaired but also pexophagy. This raises the possibility that under these circumstances either mitophagy is required for pexophagy, or that BNIP3L also directly regulates peroxisome autophagy. The latter appears to be the case.

BNIP3L is a transmembrane OMM protein, and as such we found that both endogenous and exogenous protein is almost exclusively on mitochondria under basal conditions. However, we suspected that it could also be present on peroxisomes following hypoxia. We found that BNIP3L has a dual localization signal, embedded within its transmembrane domain, and following treatment with DFP or hypoxia it becomes enriched on peroxisomes as well as mitochondria. Furthermore, we observed that mutations in the LIR domain of BNIP3L, which prevent Atg8-family protein binding, impair DFP-induced pexophagy but not BNIP3L localization on peroxisomes. Thus, as is summarized in Figure 1, our study uncovers an SLR-independent pexophagy pathway that requires the BNIP3L receptor, and, in doing so, we found that BNIP3L is a peroxisomal as well as mitochondrial protein. Though our results show that mitochondria and peroxisomes are engulfed independently by phagophores, both mitophagy and pexophagy occur at the same time.

**Figure 1.**
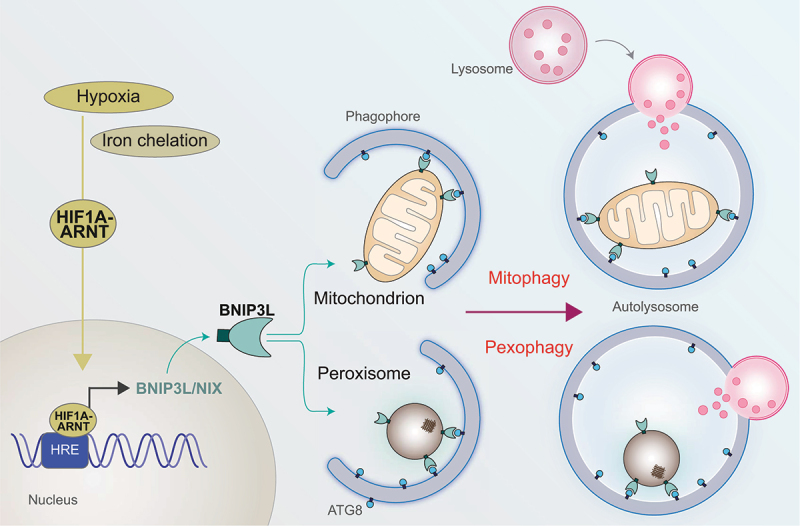
BNIP3L/NIX coordinates both mitophagy and pexophagy. Under hypoxia or iron chelation, HIF1A (hypoxia inducible factor 1 subunit alpha) is stabilized and together with ARNT/HIF1B, binds hypoxia-response elements (HREs) to drive target gene expression. One such target is the *BNIP3L* gene leading to upregulation of BNIP3L/NIX protein. BNIP3L/NIX is a mitochondrial autophagic receptor, which we now show is dually localized to peroxisomes as well as mitochondria. By interacting with autophagic Atg8-family proteins (ATG8), BNIP3L/NIX targets mitochondria and peroxisomes to forming autophagosomes. After fusion with lysosomes, both organelles are degraded.

We have shown that BNIP3L-dependent pexophagy robustly occurs in cell lines, but what about under more physiological conditions? Importantly, we were able to show that this is likely a very relevant process in vivo, as *bnip3l* knockout mouse tissue has a higher peroxisomal content. In addition, pexophagy seems to occur under the same physiological processes that activate mitophagy: during human red blood cell development and cardiomyocyte differentiation. Thus, our data demonstrate that BNIP3L regulates a physiologically significant pexophagy pathway and that an interplay exists with mitophagy.

Mitochondria and peroxisomes are key metabolic organelles and signaling pathways preserving and maintaining their function are critical for cell survival. Unsurprisingly, loss or disruption of these pathways has been associated with multiple diseases, including neurodegeneration and cancers. Impaired mitophagy is hypothesized to be a major cause of mitochondrial dysfunction, which in turn could lead to diseases such as Parkinson. Our data indicate coregulation of mitophagy and pexophagy, which are activated as a “pair” under certain conditions. Thus, the dysfunction of one could be the mirror of the dysfunction of the other and it is possible that impaired pexophagy is also contributing to mitophagy-related disease pathology. Moving forward, it could be beneficial if both pathways are analyzed in disease-related contexts. From a therapeutic point of view, our results suggest BNIP3L as an interesting target to manipulate the turnover of both mitochondria and peroxisomes.
